# *Plasmodium*-Induced Inflammation by Uric Acid

**DOI:** 10.1371/journal.ppat.1000013

**Published:** 2008-03-07

**Authors:** Jamie M. Orengo, James E. Evans, Esther Bettiol, Aleksandra Leliwa-Sytek, Karen Day, Ana Rodriguez

**Affiliations:** 1 Department of Medical Parasitology, New York University School of Medicine, New York, New York, United States of America; 2 Department of Biochemistry and Molecular Pharmacology, University of Massachusetts Medical School, Worcester, Massachusetts, United States of America; Albert Einstein College of Medicine, United States of America

## Abstract

Infection of erythrocytes with the *Plasmodium* parasite causes the pathologies associated with malaria, which result in at least one million deaths annually. The rupture of infected erythrocytes triggers an inflammatory response, which is induced by parasite-derived factors that still are not fully characterized. Induced secretion of inflammatory cytokines by these factors is considered a major cause of malaria pathogenesis. In particular, the inflammatory cytokine tumor necrosis factor (TNF) is thought to mediate most of the life-threatening pathologies of the disease. Here we describe the molecular characterization of a novel pathway that results in the secretion of TNF by host cells. We found that erythrocytes infected by *Plasmodium* accumulate high concentrations of hypoxanthine and xanthine. Degradation of *Plasmodium*-derived hypoxanthine/xanthine results in the formation of uric acid, which triggers the secretion of TNF. Since uric acid is considered a “danger signal” released by dying cells to alert the immune system, *Plasmodium* appears to have co-evolved to exploit this warning system. Identifying the mechanisms used by the parasite to induce the host inflammatory response is essential to develop urgently needed therapies against this disease.

## Author Summary

Malaria, a disease that results in more than one million deaths per year, is caused by infection with the *Plasmodium* parasite. *Plasmodium* infects erythrocytes inducing an acute inflammatory response with high fevers and elevated levels of pro-inflammatory cytokines, which contribute to the pathology of the disease. We have identified a soluble factor from infected erythrocytes that induces the production of the inflammatory cytokine TNF from murine dendritic cells. We observed that hypoxanthine is accumulated in high concentrations in Plasmodium-infected erythrocytes. Upon rupture of these erythrocytes, hypoxanthine is degraded into uric acid by a host enzyme. Uric acid is a well-known modulator of immune responses, as it is the causative agent of gout and has been identified as a danger signal for the immune system. As the malaria-induced inflammatory response contributes to most of the pathology associated with malaria infections, including death, its understanding is essential for the development of effective treatments.

## Introduction

There are over 500 million clinical malaria cases and at least one million deaths annually [1]. The pathophysiological consequences of malaria are caused by the asexual blood stage of the *Plasmodium* parasite. The rupture of infected erythrocytes triggers an inflammatory response, which is induced by parasite-derived factors that contribute to clearance of the parasite. However, the inflammatory response can also be detrimental for the host because it contributes to disease-induced pathologies. In particular, the severity of malaria has been correlated with high systemic levels of TNF [2],[3].

TNF is an endogenous, pyrogenic cytokine produced by immune cells that plays a major role in malaria pathogenesis. TNF has been implicated in the development of cerebral malaria, severe anemia and metabolic disturbances, such as acidosis and hypoglycemia [4]. Given the pathogenic downstream effects of TNF, the bioactive parasite-derived molecules released upon rupture of infected erythrocytes will likely be implicated in disease pathologies.

Two *Plasmodium*-derived molecules that induce inflammatory responses from host cells have been characterized: glycosylphosphatidylinositol (GPI) anchors [5] and a parasite-induced polymer of degraded heme called hemozoin [6]. While these molecules may contribute to the acute host pathogenesis, it is clear that other mediators of the malaria associated inflammatory response remain unknown [7].

We attempted the molecular characterization of *Plasmodium*-derived factors that trigger the release of TNF from host cells. We found that *Plasmodium*-infected erythrocytes accumulate high concentrations of the precursors of uric acid, hypoxanthine and xanthine. After erythrocyte rupture, hypoxanthine and xanthine are degraded to uric acid, which induces a strong inflammatory response from host cells, characterized by the release of TNF.

## Results

To perform the molecular characterization of TNF-inducing factors from *Plasmodium*, we used a model system in which we measured the release of TNF from cultures of murine *in vitro-*differentiated dendritic cells (DCs) in response to erythrocytes infected with the rodent malaria parasite *P. yoelii*.

**Figure 1:**
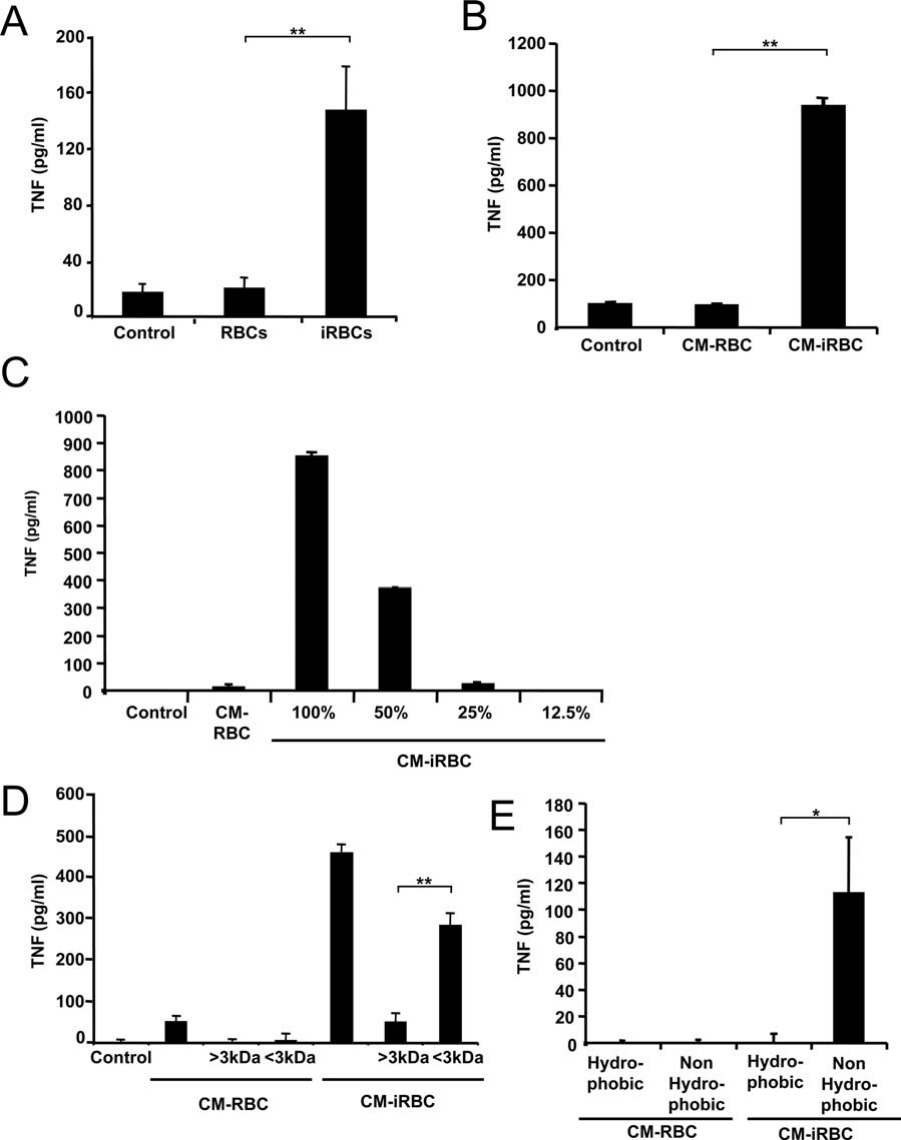
*P. yoelii*-infected erythrocytes induce TNF secretion from DCs by a soluble low MW, non-hydrophobic molecule(s). DCs were incubated with medium alone (Control), uninfected erythrocytes (RBC), *P. yoelii* infected erythrocytes (iRBC) for 6 h (A), the conditioned medium of uninfected (CM-RBC) or *P. yoelii*-infected (CM-iRBC) erythrocytes (B), either diluted in medium (C), fractionated by size (D) or hydrophobicity (E) for 24 h. TNF concentrations were determined in the incubation medium by ELISA. Data represent the average of triplicated samples with standard deviations. *P<0.05; **P<0.01.

We first determined that the release of TNF is increased from DCs incubated with *P. yoelii*-infected erythrocytes compared to DCs incubated with uninfected erythrocyte controls (Fig. 1A). We also found that the conditioned medium of *P. yoelii-*infected erythrocytes (described in Materials and Methods) induced the release of TNF from DCs, while the conditioned medium of uninfected erythrocytes did not (Fig. 1B). This effect was concentration dependent, as diluting the conditioned medium resulted in decreasing TNF release from DCs (Fig. 1C). The TNF-inducing activity was also resistant to 100°C heating and −20°C freezing (not shown). When the *P. yoelii*-conditioned medium was fractionated by size, the TNF-inducing activity was found in the fraction smaller than 3 kDa (Fig. 1D). High levels of activity were also found in the non-hydrophobic fraction of the conditioned medium of *P. yoelii*-infected erythrocytes (Fig. 1E).

**Figure 2:**
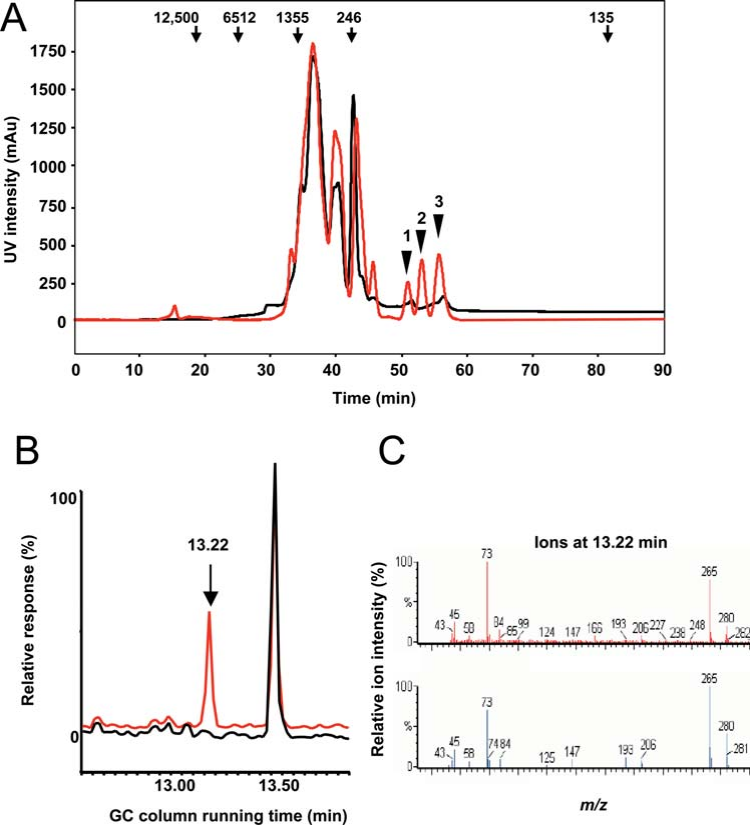
Identification of a soluble factor that induces TNF secretion by DC. (A) Ultraviolet chromatogram of the non-hydrophobic fraction from the conditioned medium of *P. yoelii* infected erythrocytes (red) compared to the non-hydrophobic fraction of the conditioned medium of uninfected erythrocytes (black) from a sizing HPLC column. Arrows indicate the position of molecular weight standards. Arrowheads point to three peaks collected for mass spectrometry analysis. (B) Total ion current plot from GC-EI-MS analysis of the TMS-derivatized fraction 1 (red) from A compared to the reagent blank (black). (C) Top, full EI-MS of the 13.22 min fraction in A. Bottom, matching mass spectrum from the NIST database identifying hypoxanthine as high confidence match.

The non-hydrophobic fraction of the conditioned medium from control and *P. yoelii*-infected erythrocytes was separated further by HPLC on a sizing column (Superdex 200 HR 10/30) monitored with a diode array ultraviolet spectrum (UV) detector. Several peaks of low molecular weight (<250 Da) were only found in the conditioned medium of *P. yoelii*-infected erythrocytes, but not in the conditioned medium of uninfected erythrocytes (Fig. 2A). Since common desalting methods exploit hydrophobicity and size to achieve separation, we were not able to isolate the low molecular weight fractions containing non-hydrophobic molecules from the HPLC buffer salts. The high concentrations of salts were toxic to DCs prohibiting the direct testing of the fractions for their activity on DCs. Thus, we identified the molecular components found in these peaks and tested their activity on DCs. Three low molecular weight peaks found only in the conditioned medium of *P. yoelii*-infected erythrocytes were isolated, subjected to trimethylsilyl (TMS) derivitization and analyzed by gas chromatography (GC) coupled with electron impact mass spectrometry (EI-MS) as previously described [8]. We detected a single GC peak at 13.22 min for peak 1 (Fig. 2B). A search of the National Institute of Standards and Technology (NIST) database of EI mass spectra returned a highly probably match with tetra-TMS hypoxanthine (Fig. 2C). The same method identified urea and xanthine as components of peaks 2 and 3 (Fig. S1). These three compounds are in the uric acid metabolic pathway [9]. Uric acid is a well-characterized pro-inflammatory molecule that in its crystallized form induces the release of inflammatory mediators [10],[11]. In addition, soluble uric acid can stimulate the production of inflammatory mediators from different cell types [12]–[14].

**Figure 3:**
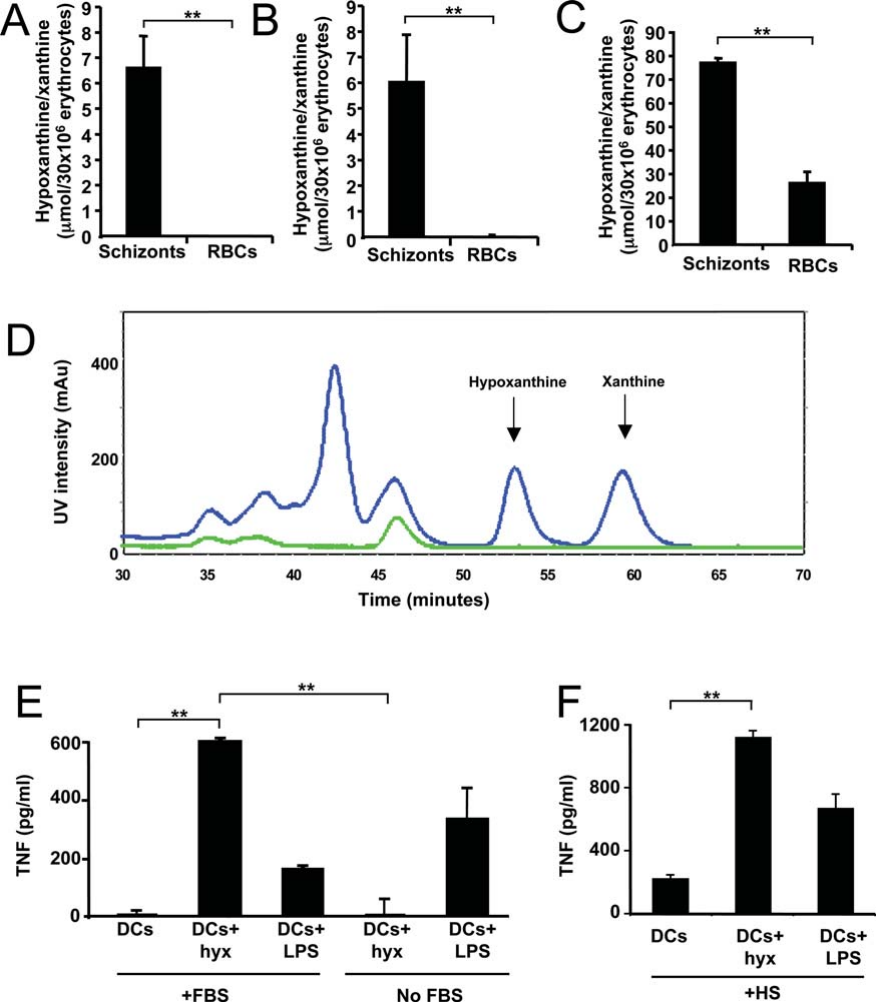
*Plasmodium* infected erythrocytes accumulate hypoxanthine/xanthine. Hypoxanthine induces DCs to secrete TNF in the presence of serum. The concentration of hypoxanthine/xanthine was determined in the soluble fraction of schizont lysates of *P. yoelii* (A), *P. berghei* (B) and *P. falciparum* (C). Lysates of uninfected murine and human erythrocytes were used as controls. (D) Ultraviolet spectrum profiles of HPLC fractionation of the soluble fraction of lysates of *P. yoelii* schizonts (blue) and uninfected erythrocytes (green). Arrows indicate the positions of hypoxanthine and xanthine when run independently. (E, F) DCs were incubated in medium supplemented or not with 10% fetal bovine (FBS; E) or human (HS; F) serum in the presence or absence of hypoxanthine or LPS for one hour. The incubation medium was removed, cells were washed, and medium supplemented with either 10% FBS or 10% HS was added. After 24 h, incubation media were collected and TNF concentrations were determined by ELISA. Data represent the average of triplicated samples with standard deviations. **P<0.01.

To confirm the MS identifications, we first determined whether hypoxanthine and xanthine are accumulated in *P. yoelii* conditioned medium (not shown) and in infected erythrocytes. We used a quantification method based on the enzyme xanthine oxidoreductase (XO), which specifically degrades hypoxanthine and xanthine to uric acid while releasing reactive oxygen species (ROS). We found that the concentration of xanthine/hypoxanthine in the soluble fraction of lysates from late stage *P. yoelii*-infected erythrocytes (schizonts) was very high compared to control uninfected erythrocytes where xanthine/hypoxanthine was not detected (Fig. 3A). Similar results were found for another rodent parasite, *P. berghei,* and for the human parasite *P. falciparum* (Fig. 3B,C). We also found that when control, human, uninfected erythrocytes were cultured *in vitro* in the presence of hypoxanthine, they too accumulated hypoxanthine, although at lower concentrations compared to *P. falciparum* infected erythrocytes. This is likely because of the excess hypoxanthine (500 µM) used in the parasite culture medium. Using HPLC sizing analysis of the soluble fraction of a lysate of *P. yoelii* schizonts, we also found two peaks in the positions corresponding to hypoxanthine and xanthine (Fig. 3D).

*Plasmodium* cannot synthesize purines de novo and imports hypoxanthine/xanthine from the extracellular environment as a purine source [15],[16]. *In vitro* the necessity of hypoxanthine for parasite growth is also well established, as supplementation of culture medium with hypoxanthine increases parasite yields three- to fourfold [17]–[20]. Likewise, the *in vitro* depletion of hypoxanthine via the addition of XO prevents parasite growth [21]. Our data suggest that the parasite imports an excess of hypoxanthine/xanthine resulting in its accumulation during the late stages of infection. Since XO activity has not been detected in erythrocytes nor in the *Plasmodium* parasite [22], imported hypoxanthine/xanthine would not be degraded into uric acid. However, upon erythrocyte rupture, the accumulated hypoxanthine/xanthine would be released into the extracellular medium where XO, which is present in the blood and intercellular fluids [23], would form uric acid producing ROS in the process.

We first determined whether hypoxanthine can induce the release of inflammatory mediators from DCs. We found that the addition of hypoxanthine to DC cultures results in TNF production, but only in the presence of bovine (Fig. 3E) or human (Fig. 3F) serum, suggesting that the processing of hypoxanthine by serum enzymes is necessary for its pro-inflammatory activities. We also confirmed that the culture medium of *P. yoelii*-infected erythrocytes only induces TNF secretion when it is incubated in the presence of serum (Fig. S2).

**Figure 4:**
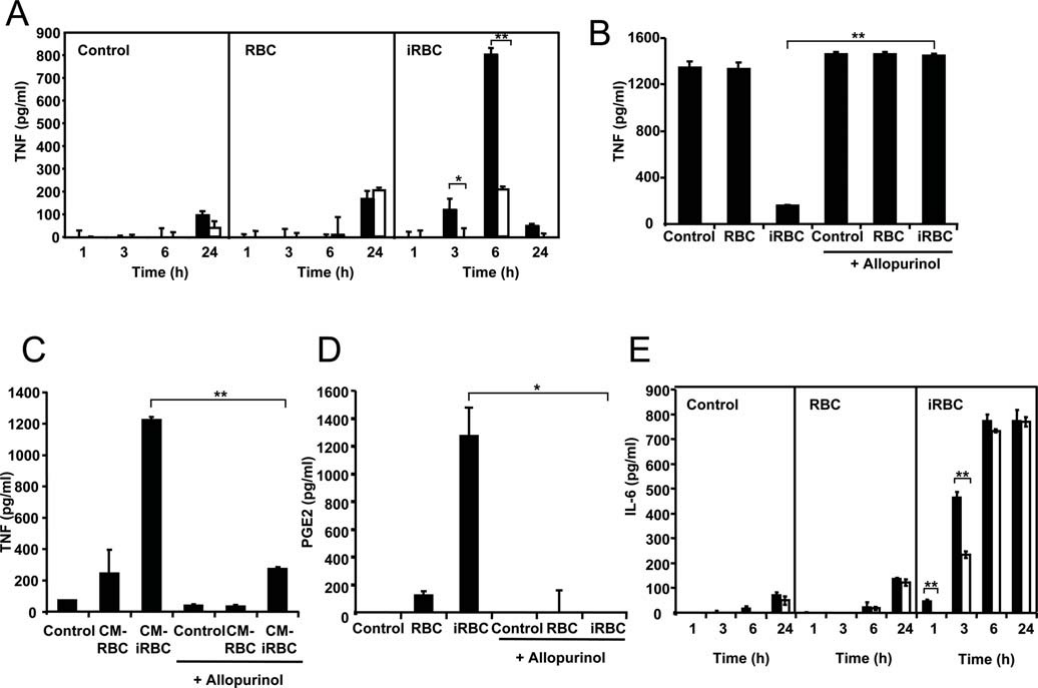
*Plasmodium*-derived hypoxanthine induces TNF, PGE_2_, and IL-6 release by DCs. (A) DCs were incubated with *P. yoelii*-infected erythrocytes alone (black bars) or in the presence of allopurinol (white bars) for the indicated times. (B) DCs were pre-incubated with media alone (Control), uninfected erythrocytes (RBCs) or *P. yoelii*-infected erythrocytes (iRBCs) for 24 h in the presence or absence of allopurinol, before addition of LPS. Incubation media was collected after additional 24 h. (C) DCs were incubated with media alone (Control), the conditioned medium of uninfected erythrocytes (CM-RBC), or the conditioned medium of *P. yoelii*-infected erythrocytes (CM-iRBC) in the presence or absence of allopurinol for 24 h. (D) DCs were pre-incubated with media alone (Control), uninfected erythrocytes (RBCs) or *P. yoelii*-infected erythrocytes (iRBCs) for 24 h in the presence or absence of allopurinol. (E) DCs were incubated with *P. yoelii*-infected erythrocytes alone (black bars) or in the presence of allopurinol (white bars) for the indicated times. Incubation media were collected and TNF (A–C), PGE_2_ (D) or IL-6 (E) concentrations were determined by ELISA. Data represent the average of triplicated samples with standard deviations. *P<0.05; **P<0.01.

To study if the release of pro-inflammatory mediators by DCs can be induced by *P. yoelii*-derived hypoxanthine/xanthine, DCs were incubated with *P. yoelii*-infected erythrocytes in the presence of allopurinol, an inhibitor of XO that prevents the formation of uric acid from hypoxanthine or xanthine [24]. Allopurinol has toxic effects on trypanosomatids [25],[26], but this drug does not inhibit the growth of *Plasmodium* [27],[28]. First we analyzed the effects of allopurinol on the release of TNF induced by *P. yoelii*-infected erythrocytes from DCs. We observed that TNF, which was released by DCs at 3 and 6 h after the addition of infected erythrocytes, was inhibited almost completely by allopurinol (Fig. 4A). This suggests that the degradation of *Plasmodium*-derived hypoxanthine/xanthine into uric acid is a major contributor to the release of TNF by DCs. We also observed that TNF was no longer secreted by DCs 24 h after addition of infected erythrocytes (Fig. 4A). At this time, the addition of another TNF-inducing stimulus, such as LPS, did not activate TNF secretion by DCs, most likely due to tolerance induced by the initial parasite activation (Fig. 4B). In the presence of allopurinol, DCs secrete TNF in response to LPS even after incubation with infected erythrocytes (Fig. 4B). This is probably because the initial TNF response induced by the parasite and the subsequent tolerogenic effect are inhibited by allopurinol. Hypoxanthine degradation could therefore contribute to the *Plasmodium*-induced tolerance to endotoxin-mediated activation that is observed both in malaria patients and in *in vitro* assays [29]. We also found that inhibiting hypoxanthine degradation with allopurinol inhibited TNF secretion induced by the conditioned medium of *P. yoelii*-infected erythrocytes (Fig. 4C). As a control, we confirmed that addition of allopurinol did not inhibit the secretion of TNF induced by LPS (Fig. S3).

We also determined the role of hypoxanthine degradation in the induction of two inflammatory mediators that are released by DCs in response to *Plasmodium*: prostaglandin E_2_ (PGE_2_) [30] and interleukin (IL)-6 [31]. Allopurinol inhibited *P. yoelii*-infected erythrocyte induced release of PGE_2_ by DCs (Fig. 4D) and the early DC release of IL-6 (Fig. 4E).

**Figure 5:**
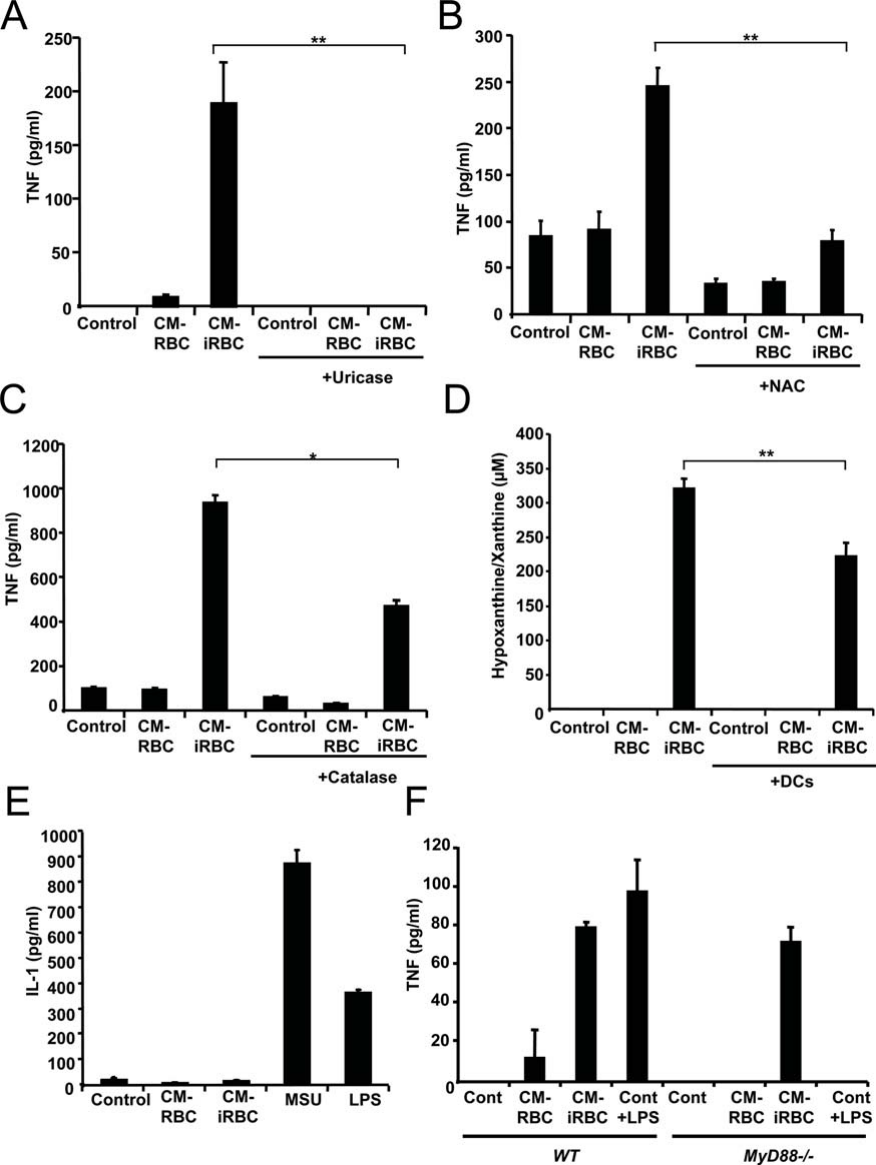
Uric acid induces TNF release by DCs via a MyD88 independent pathway. (A–C) DCs were incubated with medium alone (Control), the conditioned medium of uninfected erythrocytes (CM-RBCs), or the conditioned medium of *P. yoelii*-infected erythrocytes (CM-iRBCs) cultivated in the presence of 0.1 mg/ml uricase, 30 mM NAC or 100 U/ml catalase for 24 h. (D) Hypoxanthine concentrations were measured in the medium alone (Control), the conditioned medium of uninfected erythrocytes (CM-RBCs), or the conditioned medium of *P. yoelii-*infected erythrocytes (CM-iRBCs) before and after incubation with DCs in medium with serum for 24 h. (E) DCs were incubated with media alone (Control), the conditioned medium of uninfected (CM-RBC) or *P. yoelii*-infected (CM-iRBC) erythrocytes, 1 mg/ml monosodium urate crystals (MSU) or LPS for 24 h. (F) DCs from wild type (WT) or *MyD88-/-* mice were incubated with media alone (Control) the conditioned medium of uninfected (CM-RBC) or *P. yoelii*-infected (CM-iRBC) erythrocytes for 24 h. (A–C, E–F) Incubation media were collected and TNF or IL-1 concentrations were determined by ELISA. Data represent the average of triplicated samples with standard deviations. *P<0.05; **P<0.01.

When uricase, a specific enzyme that degrades uric acid, was added to the conditioned medium of *P. yoelii*-infected erythrocytes, TNF release by DCs was inhibited, confirming the role of uric acid in the observed inflammatory response (Fig. 5A). As control, we confirmed that addition of uricase did not inhibit the secretion of TNF induced by LPS (Fig. S3).

Reactive oxygen species (ROS), generated when hypoxanthine is degraded into uric acid, can also activate DCs [32]. We therefore tested the effects of the antioxidant N-acetylcysteine (NAC) and catalase, an enzyme that degrades hydrogen peroxide and neutralizes its inflammatory effect. We observed that NAC (Fig. 5B) and catalase inhibit the secretion of TNF by DCs (Fig. 5C), indicating that ROS contribute to the inflammatory effect of uric acid. As a control, we confirmed that heat-inactivated catalase did not have an effect on DC production of TNF (Fig. S4).

To confirm that hypoxanthine is being degraded to uric acid in our system, we measured the concentration of hypoxanthine present in the conditioned medium of *P. yoelii*-infected erythrocytes before and after incubation with DCs. We found a decrease in hypoxanthine/xanthine of approximately 100 µM (Fig. 5D). We also found accumulation of uric acid in this medium, however, to a much lower concentration (13 µM). This is expected, since the presence of uricase in the serum probably prevents the accumulation of uric acid in the culture medium.

Under these conditions, it is not likely that uric acid crystals are formed in the culture medium, since the threshold for crystallization of uric acid in biological fluids is (100 µg/ml) [33]. We also did not find uric acid crystals in the culture medium of DCs and the conditioned medium of *P. yoelii*-infected erythrocytes or in DCs that had phagocytosed infected erythrocytes. Thus, it appears that the mechanism activating TNF release by DCs in our conditions is not classical uric acid crystal formation, but a more complex stimulus involving at least two components: soluble uric acid and ROS formed as a result of *Plasmodium*-derived hypoxanthine degradation.

Uric acid crystals activate the NALP3 inflammasome, resulting in the production of active IL-1 [34]; however, the receptor and the molecular mechanism that triggers TNF release remains unknown [35]. We found that the conditioned medium of *P. yoelii*-infected erythrocytes did not induce the release of IL-1, while monosodium urate crystals did (Fig. 5E), suggesting again that the mechanism inducing TNF release by *Plasmodium* is different from the formation of urate crystals.

To determine whether the *Plasmodium-*derived uric acid induction of TNF from DCs through a MyD88 dependent pathway, the conditioned medium of *P. yoelii*-infected erythrocytes was incubated with DCs derived from wild type or *MyD88-/-* mice. We found that the conditioned medium induces TNF via a MyD88 independent pathway (Fig. 5F). Accordingly, it has been shown that the monocyte component of the inflammatory response to monosodium urate crystals is MyD88-independent [36]. Our result also confirms that the release of TNF observed in our system is not mediated by any of the characterized *Plasmodium* inflammatory mediators: GPI and hemozoin, which activate TLR-MyD88 dependent responses [37].

## Discussion

Uric acid has been identified as a danger signal released from necrotic cells that activates DCs [8]. It was shown that crystalline uric acid activates DCs. Since the physiological concentrations of uric acid (40–60 µg/ml) are near-saturating, crystallization probably occurs when an excess is released during cellular damage caused by infection or some other stress [8]. Excessive concentrations of uric acid are also the causative agent of gout, which develops as a result of crystal formation in the joints. This causes the release of pro-inflammatory mediators (such as TNF) from different immune cell types [10].

Elevated levels of uric acid are observed in the blood of malaria-infected humans and mice [38]–[40]. This could be caused by renal dysfunction induced by infection [41], but may be also caused by increased hypoxanthine degradation in the blood. Most mammals, such as mice, have uricase, a liver enzyme that degrades uric acid [42]. The presence of this enzyme affords these mammals low serum uric acid levels (5–20 µg/ml) [43]. However, in humans uric acid is the final product of hypoxanthine degradation because the uricase enzyme was mutated throughout evolution and its activity cannot be found in human serum [43]. Thus, humans have higher serum uric acid levels (40–60 µg/ml) and a lower capacity to control increases in uric acid, which may result in more prominent role of this pathway in the inflammatory response to malaria in humans.

High concentrations of soluble uric acid induce the release of inflammatory mediators from different cell types [12]–[14], suggesting that the soluble uric acid formed via hypoxanthine degradation could also contribute to the malaria induced inflammatory response. We were unable to observe uric acid crystals either *in vitro* in the culture medium of *P. yoelii*-infected erythrocytes or *in vivo* in infected mice. Our results suggest that the release of TNF that we observed from murine DCs is not dependent on the formation of crystals and appears to be mediated by soluble uric acid and reactive oxygen species.

During *Plasmodium* infections in humans, elevated levels of uric acid could facilitate crystallization in localized environments, since the uric acid concentration in the blood of patients with severe malaria [38] can reach levels close to the threshold of uric acid crystallization in biological fluids (100 µg/ml) [33]. Infected erythrocytes concentrate in specific organs, such as the spleen, where they are also found in the extra-vascular tissue [44]. It is possible that this environment could also facilitate crystallization, as high local concentrations of uric acid above the threshold of crystallization might easily be reached upon schizont rupture and hypoxanthine release in these organs, especially in synchronized infections. Uric acid may also be formed in the brain when infected erythrocytes are sequestered in capillaries, thus forming a micro-environment that would facilitate crystallization. Interestingly, elevated uric acid levels are found in the brains of mice infected with *P. berghei*, although this increase is not associated with the development of experimental cerebral malaria in mice [45]. *Plasmodium*-derived hypoxanthine must also be converted to uric acid in the phagosome of DCs and macrophages upon uptake and degradation of infected erythrocytes, since they express XO [24]. Within this localized environment, high concentrations of uric acid would easily be reached.

The degradation of hypoxanthine/xanthine into uric acid by XO results in the formation of ROS, which can induce the release of inflammatory mediators from immune cells [46]. We found that the antioxidant NAC and the degradation of hydrogen peroxide by catalase result in decreased secretion of TNF, suggesting that ROS contribute to the inflammatory effect of uric acid. ROS production by hypoxanthine degradation may also contribute to the increased oxidative stress observed during malaria infection, which also plays a role in the associated pathologies [47].

When patients with acute complicated malaria due to *P. falciparum* infection were treated with an anti-malarial drug in combination with allopurinol, they had a faster decrease in fever and splenomegaly compared to controls treated with the anti-malarial alone [28], suggesting that hypoxanthine degradation plays a role in the general inflammatory response during *P. falciparum* infection.

In this study we characterize a novel pathway induced by *Plasmodium* that leads to the secretion of inflammatory mediators by DCs. Since most of the pathologies associated with malaria are caused by the inflammatory response of the host [48], identification of the mechanisms used by the parasite is essential to develop new therapeutic strategies against this disease. Since an adequate cytokine balance is important for resolving *Plasmodium* infections without severe pathology [49], a detailed characterization of the effects of uric acid in the malaria associated pathologies is required to determine the possible applications of this finding.

## Materials and Methods

### Reagents, mice and parasites

All chemical reagents were from Sigma unless otherwise specified. Parasites used were: *P. yoelii* 17X NL, *P. berghei* ANKA and *P. falciparum* 3D7. Female, 6–8 week old Swiss Webster (SW) or BALB/c mice were purchased from Taconic (Germantown, NY). Control C57/BL6 WT and *MyD88-/-* mice were kindly provided by Dr. Maria Mota (University of Lisbon, Portugal). Uricase (Elitek, Sanofi-Aventis) and catalase from mouse liver (Sigma) were further purified using EndoClean gel^TM^ filtration columns (BioVintage). The activity for catalase is 897 Units/mg of protein. Monosodium urate crystals were prepared as described [8].

### Isolation of erythrocytes

Blood was isolated from *P. yoelii-*infected or uninfected anesthetized SW mice via cardiac puncture and diluted in 100 U heparin in PBS. Erythrocytes were washed three times with PBS and separated from plasma and white blood cells by centrifugation at 1,800 g for 5 minutes. The layer of white blood cells that sediments on the top of the erythrocytes was removed. Blood was either used as prepared or further processed depending on the experiment.

### Separation of schizonts from *P. yoelii* and *P. berghei*-infected blood

Washed infected erythrocytes were separated into schizont and non-schizont stages by centrifugation using a 53% Accudenz (Accurate Chemical and Scientific Corp, Westbury, NY) density gradient solution in PBS. Erythrocytes from a matched non-infected mouse were treated in the same way. The purified schizonts were centrifuged at 1,800 g for 5 minutes and washed 2 times with PBS.

### Preparation of the conditioned medium of *P. yoelii*-infected erythrocytes

To standardize parasite load, the conditioned medium consisted of 30% schizonts and 70% of the non-schizont stages from the gradient described above. In parallel, control conditioned medium was prepared using erythrocytes from an uninfected mouse. To mimic schizont lysis occurring in the infected conditioned medium, 30% of the erythrocytes from the uninfected mouse were osmotically lysed and combined with uninfected erythrocytes to make the control conditioned medium. Erythrocytes were plated at a density of 5×10^8^/ml and incubated in medium without serum (D-MEM, 100 U/ml penicillin, 100 µg/ml streptomycin, 2 mM L-glutamine) for 48 h at 37°C, 5% CO_2_. Cultures were centrifuged at 1800 g for 5 min to collect the supernatant. This supernatant was heated to 100°C for 5 min, centrifuged at 1,800 g for 5 min and filtered through a 0.2 µm diameter filter.

### Incubation of DCs with hypoxanthine, *P. yoelii*-infected erythrocytes or conditioned medium and the quantification of cytokines

Murine myeloid bone marrow-derived DCs were differentiated as previously described [50]. Briefly, female, BALB/c mice were euthanized and femurs and tibias were removed aseptically. Bone marrow was flushed using a syringe with a 28G1/2 inch. Bone marrow precursors were cultured on Petri dishes for 7–10 days at 37°C, 5% CO_2_ in the presence of 30% conditioned medium from the myeloma cell line Ag8653 that expresses the mouse recombinant granulocyte monocyte-colony stimulating factor (GM-CSF) [51]. Conditioned medium contained 10–20 mg/ml of GM-CSF. For the experiments, DCs (10^6^ cells/ml) were incubated with either medium alone or the conditioned medium of uninfected or *P. yoelii*-infected erythrocytes supplemented with 10% FBS and 5% GM-CSF supernatant. DCs were also incubated with uninfected or *P. yoelii*-infected erythrocytes at a 1∶100 ratio (DCs:erythrocytes) using 30% schizonts and 70% non-schizont mixtures in medium supplemented with 10% FBS and 5% GM-CSF supernatant for 24 h at 37°C, 5% CO_2_. When indicated, allopurinol (2 mM), lipopolysaccharide (LPS) from *Salmonella typhimurium* (1 µg/ml), N-acetylcysteine (NAC) (30 mM), Catalase (100 U/ml) or Uricase (0.1mg/ml) was added. DCs were also incubated with hypoxanthine (3 mM) for 1 h in the presence or absence of serum. The cells were then washed and incubated for 24 h in medium with 10% FBS or human serum, as indicated. Incubation media from DCs/erythrocytes co-cultures was collected and assayed by ELISA for the secretion of TNF (OPTI-EIA kit, BD Biosciences).

### Characterization and molecular identification of the *P. yoelii* active molecules

#### Fractionation of the conditioned media

The conditioned media of uninfected and *P. yoelii*-infected erythrocytes were fractionated using either Centricon® filters with a membrane size cut off of 3 kDa by centrifugation at 1,800 g for 90 minutes or Sep-Pak® reverse phase columns that retain hydrophobic molecules. The fraction not retained by the column containing non-hydrophobic molecules was dried, reconstituted at 10× concentration in DMEM without salts and used for chromatographic analysis.

#### Chromatographic analysis

Chromatographic separations were performed using a Waters HPLC model 2790 system. The non-hydrophobic, concentrated fractions of the conditioned medium of uninfected and *P. yoelii*-infected erythrocytes were purified on a Superdex Peptide 10/300 GL column. The buffer used was 0.02 M phosphate buffer, 0.25 M NaCl, pH 7.2 with a flow rate of 0.5 ml/min at room temperature. Unique peaks were collected, precipitated by addition of 5 volumes of acetone and dried. Molecular identification was performed as described by Shi, et al. [8]. Trimethylsilyl (TMS) derivatives were formed by the addition of 25 µl of silylating reagent (N,O-bis-(trimethylsilyl)trifluoroacetamide, pyridine, hexamethyldisilazane and trimethylchlorosilane;13∶2∶1∶10 v/v) followed by heating at 100°C for 30 min. The samples (2 µl) were then analyzed by GC-MS using a Waters Quattro-II triple quadrupole GC-MS system with a DB-5 (30 m, 0.25 mm i.d., 0.25 µm phase thickness) fused silica capillary column (J&W Scientific) in the splitless injection mode with He as the carrier gas at 0.9 ml/min. The column temperature was programmed from 80°C to 300°C at 10°C/min. The injector and transfer lines were maintained at 300°C. Positive ion electron impact ionization was used with the source temperature at 200°C and the ionization energy at 70 eV. Full mass spectra were acquired from m/z 40 to 800 at 1-second intervals. Spectra were compared to the NIST database for compound identification.

#### *In vitro P. falciparum* culture and harvesting

*In vitro* cultures of erythrocytic asexual stages of the *P. falciparum* strain 3D7 were maintained in culture medium (RPMI 1640, 25 mM HEPES, 10 µg/ml gentamycin, 0.5 mM hypoxanthine, pH 6.75), supplemented with 25 mM sodium bicarbonate and 10% human serum. Cultures were maintained at 5% hematocrit (o+, sickle cell negative, leukocyte depleted erythrocytes) in atmospheric conditions of 1% oxygen, 5% carbon dioxide and 94% nitrogen. Culture medium was changed daily and cultures were routinely sub-cultured to maintain a parasitemia of less than 6%. Parasitemia's were measured daily and determined by counting the number of parasitized erythrocytes from 500 cells in a Giemsa stained thin blood smear. Late stage trophozoites and schizont infected erythrocytes were harvested by the gelatine flotation method [52]. Gelatine and wash solutions did not contain hypoxanthine. Non-parasitized erythrocytes were maintained under the same conditions and used as a control.

### Preparation of the soluble fraction of *Plasmodium* lysates

Schizonts of *P. yoelii*, *P. berghei* or *P. falciparum* were purified as described above. As a control for the murine parasites, uninfected blood from age, sex, and strain-matched mice was harvested in parallel. As a control for *P. falciparum*, non-parasitized erythrocytes were prepared in parallel. Erythrocytes (1×10^8^ cells/ml) were subjected to 10 cycles of freeze/thawing. Lysates were centrifuged at 10,000 g for 10 min and supernatant was collected.

### Assay for hypoxanthine/xanthine in *Plasmodium* lysates

To detect xanthine/hypoxanthine concentrations in the soluble fraction of erythrocyte lysates, the Amplex Red® Xanthine/Xanthine Oxidase Assay Kit was used according to the manufacturer's instructions. Briefly, each reaction contained 50 µM Amplex Red reagent, 0.2 U/ml horseradish peroxidase, 20 mU/ml xanthine oxidase and the soluble fraction of either schizont lysates or uninfected RBC lysates. Commercial hypoxanthine at different concentrations was used as a standard control. The reactions were incubated for 30 min at 37°C and fluorescence was measured in a microplate reader using excitation at 530 nm and emission at 590 nm. Background fluorescence was subtracted from each data point.

## Supporting Information

Figure S1 Identification of fractions 2 and 3. (A,C) Total ion current plot from GC-EI-MS analysis of the TMS-derivatized fractions 2 (A) and 3 (C) from Fig. 2 (red) compared to the reagent blank (black). (B,D) Top, full EI-MS of the 6.36 minute fraction in A and 15.40 minute fraction in C. Bottom, matching mass spectrum from the NIST database identifying urea (B) and xanthine (D) as high confidence matches.
(2.36 MB TIF)

Figure S2 Conditioned medium requires serum to induce TNF secretion by DCs. DCs were incubated with media alone (Control), the conditioned medium of uninfected (CM-RBC) or *P. yoelii*-infected (CM-iRBC) erythrocytes for 1 h in the presence or absence of 10%FBS. The incubation medium was removed, cells were washed and medium supplemented with 10% FBS was added. After 24 h, incubation media were collected and TNF concentrations were determined by ELISA. Data represent the average of triplicated samples with standard deviations.
(1.12 MB TIF)

Figure S3 Uricase and allopurinol do not inhibit TNF secretion induced by LPS. DCs were incubated with media alone (Control) or with 1 µg/ml LPS in the presence of 0.1 mg/ml Uricase or 2 mM Allopurinol for 24 h. Incubation media were collected and TNF concentrations were determined by ELISA. Representative results from one of at least two independent experiments are shown. Error bars indicate standard deviation of triplicate samples.
(1.05 MB TIF)

Figure S4 Heat-inactivated catalase does not inhibit TNF secretion induced by the conditioned medium. DCs were incubated with media alone (Control) or the conditioned medium of *P. yoelii*-infected erythrocytes (CM-iRBCs) in the presence or absence of 100 U/ml heat-inactivated Catalase for 24 hrs. TNF concentrations were determined in the incubation medium by ELISA. Representative results from one of at least two independent experiments are shown. Error bars indicate standard deviation of triplicate samples.
(1.03 MB TIF)
