# Supplementary figures and images for: Plasmodium-Induced Inflammation by Uric Acid
Source: PLoS Pathog. 2008 Mar 7;4(3):e1000013. doi: 10.1371/journal.ppat.1000013 (PMC2267007; doi:10.1371/journal.ppat.1000013)

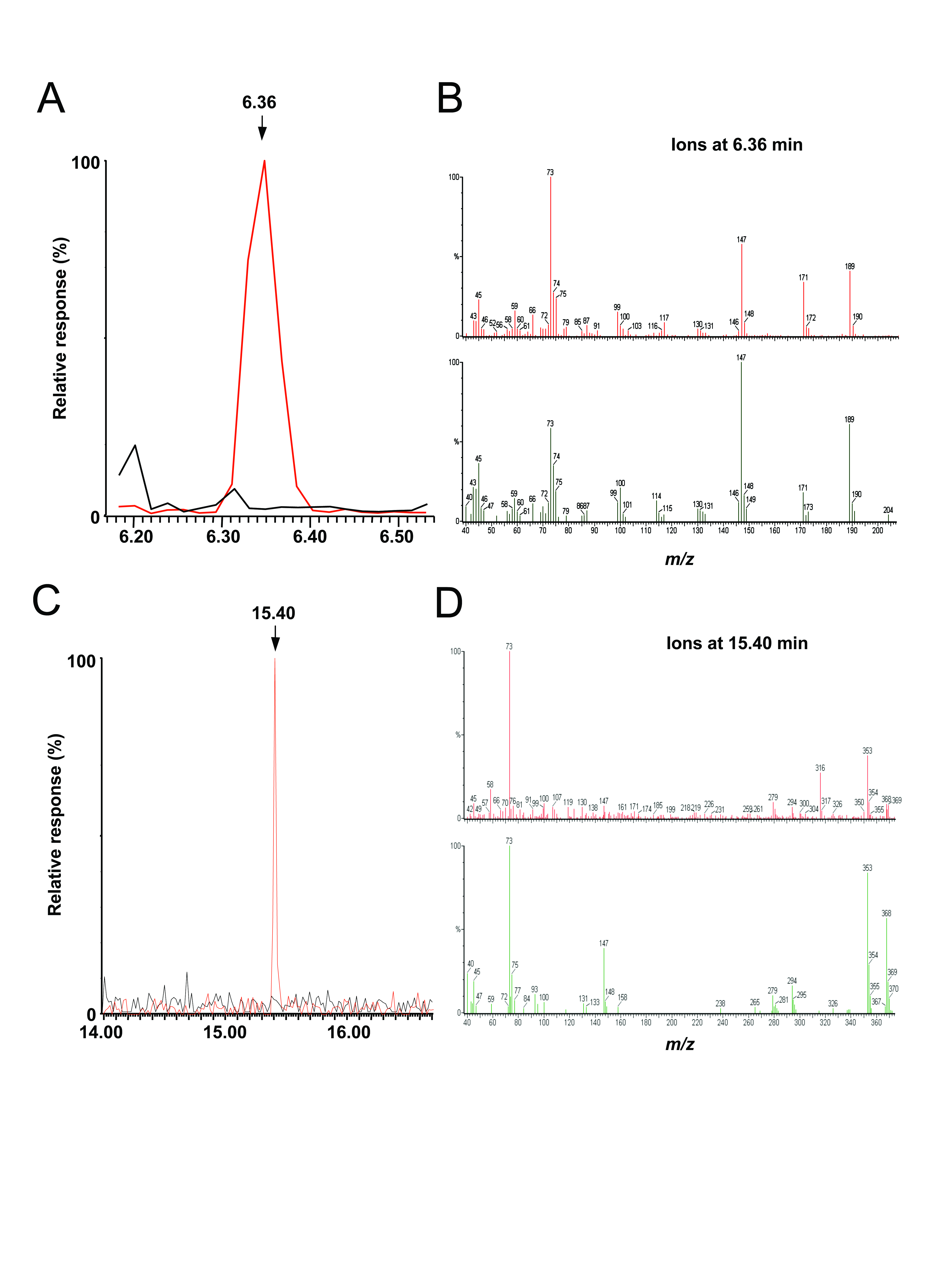

Supplement: Figure S1 — Identification of fractions 2 and 3. (A,C) Total ion current plot from GC-EI-MS analysis of the TMS-derivatized fractions 2 (A) and 3 (C) from Fig. 2 (red) compared to the reagent blank (black). (B,D) Top, full EI-MS of the 6.36 minute fraction in A and 15.40 minute fraction in C. Bottom, matching mass spectrum from the NIST database identifying urea (B) and xanthine (D) as high confidence matches. (2.36 MB TIF) [file ppat.1000013.s001.tif]

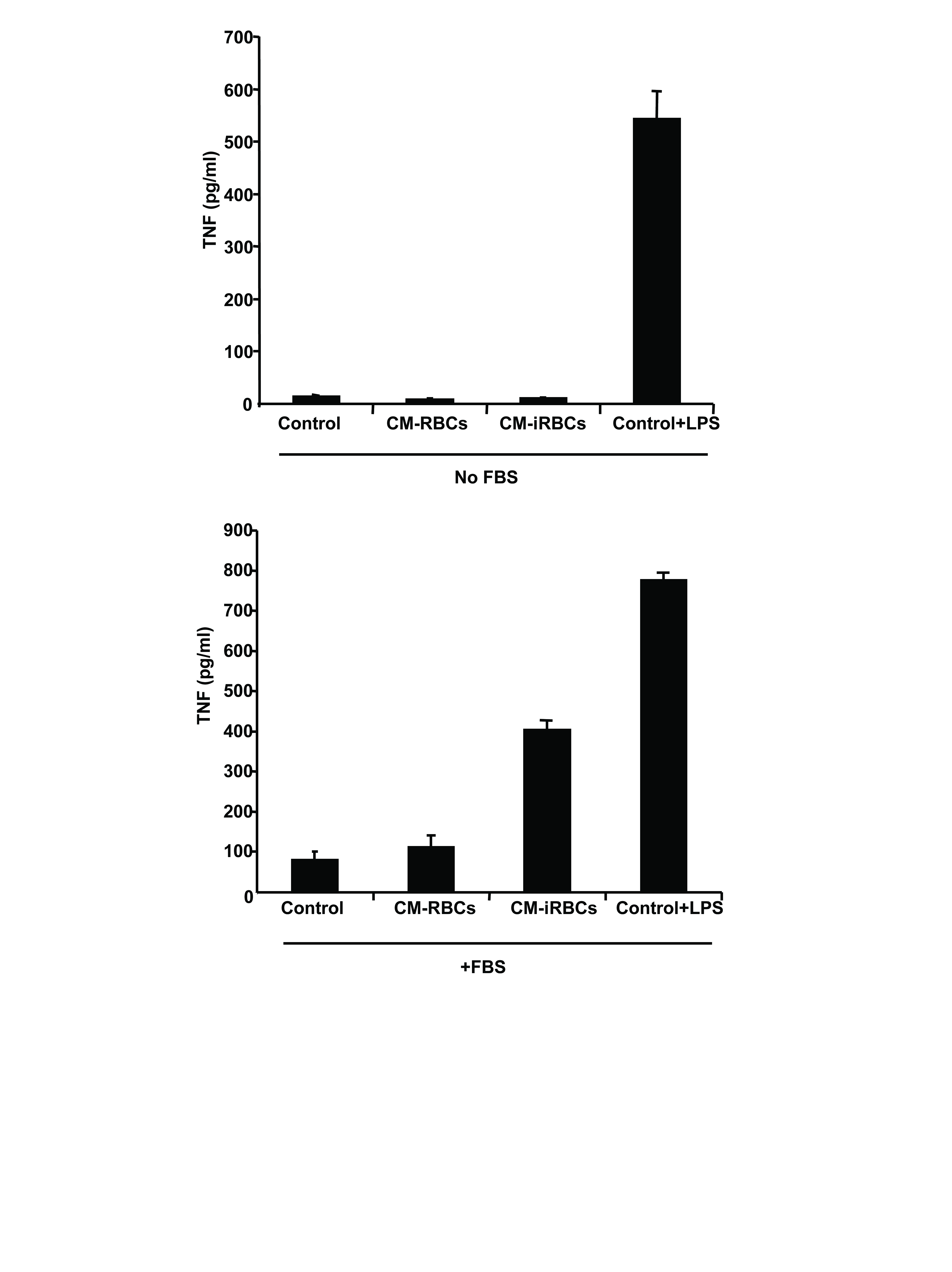

Supplement: Figure S2 — Conditioned medium requires serum to induce TNF secretion by DCs. DCs were incubated with media alone (Control), the conditioned medium of uninfected (CM-RBC) or P. yoelii-infected (CM-iRBC) erythrocytes for 1 h in the presence or absence of 10%FBS. The incubation medium was removed, cells were washed and medium supplemented with 10% FBS was added. After 24 h, incubation media were collected and TNF concentrations were determined by ELISA. Data represent the average of triplicated samples with standard deviations. (1.12 MB TIF) [file ppat.1000013.s002.tif]

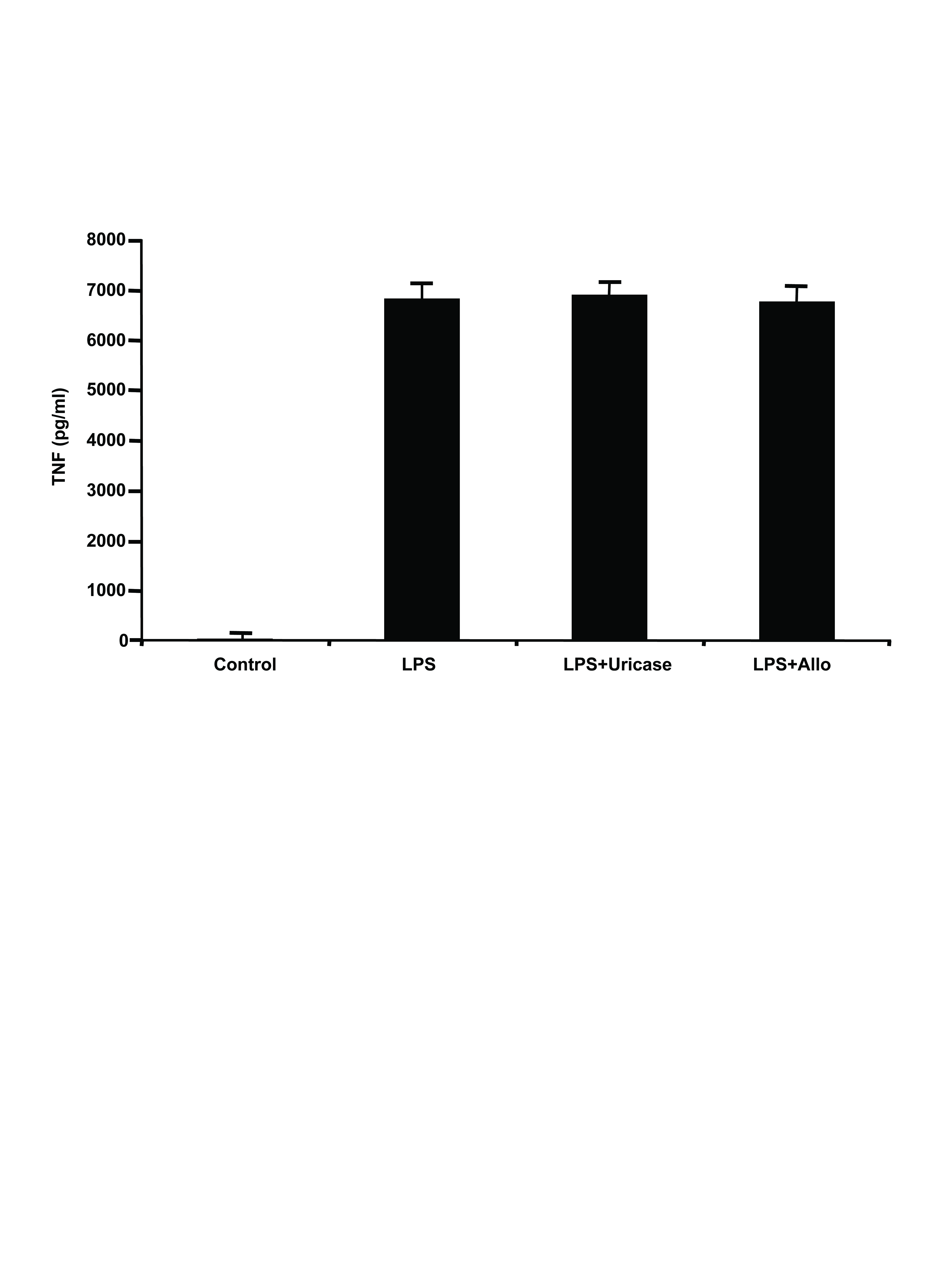

Supplement: Figure S3 — Uricase and allopurinol do not inhibit TNF secretion induced by LPS. DCs were incubated with media alone (Control) or with 1 µg/ml LPS in the presence of 0.1 mg/ml Uricase or 2 mM Allopurinol for 24 h. Incubation media were collected and TNF concentrations were determined by ELISA. Representative results from one of at least two independent experiments are shown. Error bars indicate standard deviation of triplicate samples. (1.05 MB TIF) [file ppat.1000013.s003.tif]

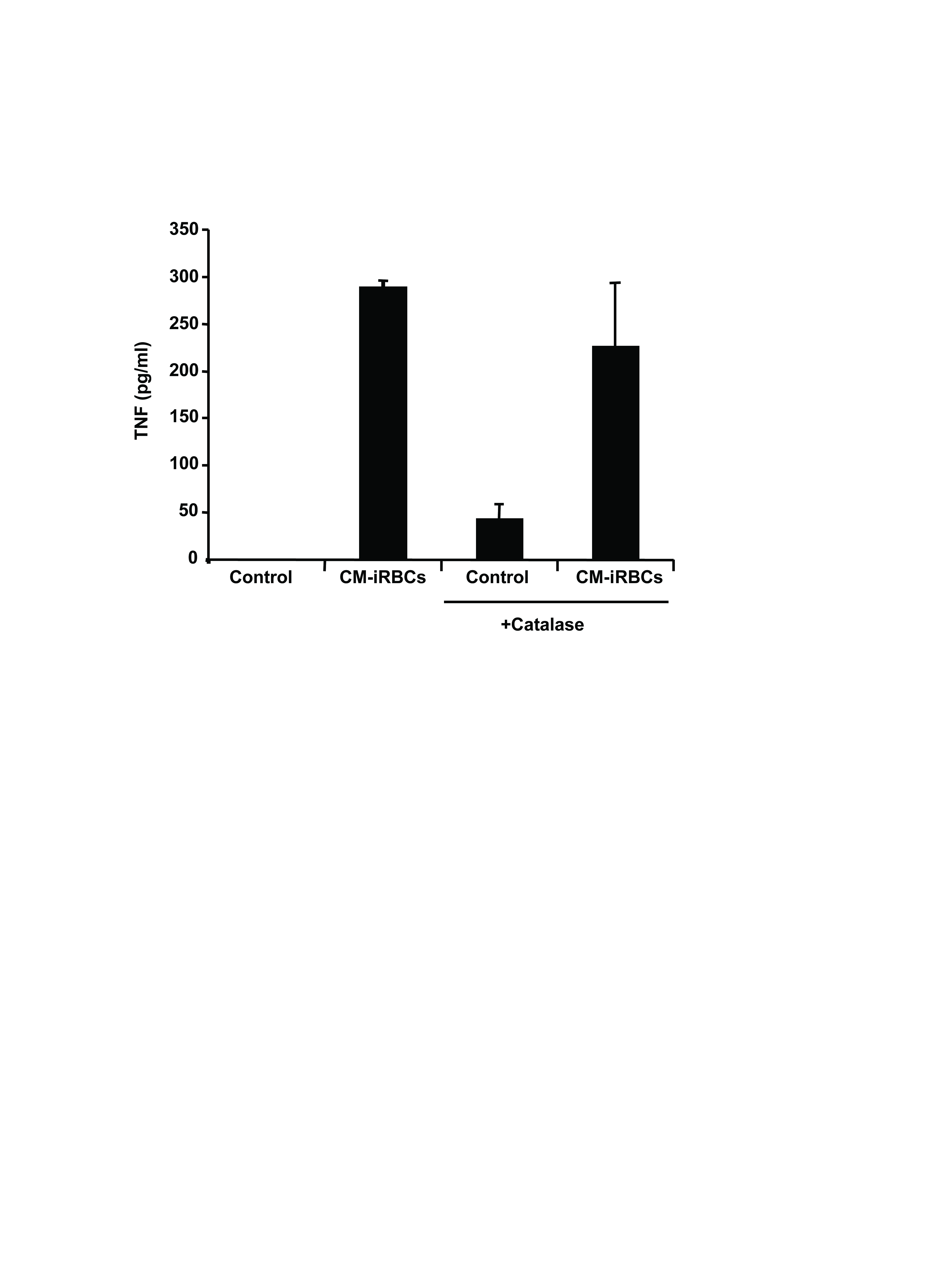

Supplement: Figure S4 — Heat-inactivated catalase does not inhibit TNF secretion induced by the conditioned medium. DCs were incubated with media alone (Control) or the conditioned medium of P. yoelii-infected erythrocytes (CM-iRBCs) in the presence or absence of 100 U/ml heat-inactivated Catalase for 24 hrs. TNF concentrations were determined in the incubation medium by ELISA. Representative results from one of at least two independent experiments are shown. Error bars indicate standard deviation of triplicate samples. (1.03 MB TIF) [file ppat.1000013.s004.tif]
